# Acceptance and adoption of Lean redesigns in primary care: A contextual analysis of implementation among frontline providers

**DOI:** 10.1186/1748-5908-10-S1-A65

**Published:** 2015-08-20

**Authors:** Dorothy Hung, Caroline Gray, Meghan Martinez, Michael Harrison

**Affiliations:** 1 Palo Alto Medical Foundation Research Institute, Mountain View, CA 94040, USA; 2 Agency for Healthcare Research and Quality, Rockville, MD 20850, USA

## 

This study funded by AHRQ examines the implementation and scale-up of Lean methodology for enhancing value in a large, ambulatory care delivery system. While evidence indicates that Lean techniques can lead to higher quality care at lower cost, its success or failure is inextricably tied to the views and activities of frontline care providers who are the daily implementers of intervention components. This study explores how such views and activities impacted system-wide efforts to redesign primary care.

Our analysis was guided by a modified version of the Consolidated Framework for Implementation Research, which articulates various "measures" of success when implementing process redesigns. Drawing on over 100 in-depth interviews with physicians and staff, we sought to understand the extent to which new Lean workflows were accepted and adopted into practice, and the contextual factors that impacted implementation success.

Frontline perceptions of Lean's potential to enhance value were impacted in part by: local dynamics of the care team; perceived skill or competency of team members (namely, medical assistants and licensed vocational nurses) in taking on new roles or scopes of work; and physicians' own perceived efficiency, or lack thereof, prior to the introduction of Lean redesigns. The implementation strategy used by the organization was also critical. Physicians and staff who viewed the effort as "top-down" or "inflexible" were less likely to comply with changes. Even those who expressed positive views about Lean as an overall strategy for redesigning care, but who found the implementation process excessively top-down, were less likely to adopt more efficient workflows.

Gaining "buy-in" from frontline providers is critical to implementing care processes that are designed to improve the delivery of health care. Understanding how clinical insiders' views inform their decision to embrace or reject changes may be instructive for organizations attempting to implement similar initiatives.

